# Drug-resistant tuberculosis in HIV-infected patients in a national referral hospital, Phnom Penh, Cambodia

**DOI:** 10.3402/gha.v8.25964

**Published:** 2015-01-22

**Authors:** Genevieve Walls, Sophie Bulifon, Serge Breysse, Thol Daneth, Maryline Bonnet, Northan Hurtado, Lucas Molfino

**Affiliations:** 1 Médecins Sans Frontières (MSF) France, Phnom Penh, Cambodia; 2 Department of Infectious Diseases, Middlemore Hospital, Auckland, New Zealand; 3 AP-HP, Service de Pneumologie, CHU Bicêtre, Paris, France; 4 Epicentre, Paris, France; 5 Médecins Sans Frontières (MSF) France, Paris, France

**Keywords:** tuberculosis, drug resistant, HIV, Cambodia

## Abstract

**Background and objective:**

There are no recent data on the prevalence of drug-resistant tuberculosis (DR TB) in Cambodia. We aim to describe TB drug resistance amongst adults with pulmonary and extra-pulmonary TB and human immunodeficiency virus (HIV) co-infection in a national referral hospital in Phnom Penh, Cambodia.

**Design:**

Between 22 November 2007 and 30 November 2009, clinical specimens from HIV-infected patients suspected of having TB underwent routine microscopy, *Mycobacterium tuberculosis* culture, and drug susceptibility testing. Laboratory and clinical data were collected for patients with positive *M. tuberculosis* cultures.

**Results:**

*M. tuberculosis* was cultured from 236 HIV-infected patients. Resistance to any first-line TB drug occurred in 34.7% of patients; 8.1% had multidrug resistant tuberculosis (MDR TB). The proportion of MDR TB amongst new patients and previously treated patients was 3.7 and 28.9%, respectively (*p*<0.001). The diagnosis of MDR TB was made after death in 15.8% of patients; in total 26.3% of patients with MDR TB died. The diagnosis of TB was established by culture of extra-pulmonary specimens in 23.6% of cases.

**Conclusions:**

There is significant resistance to first-line TB drugs amongst new and previously treated TB–HIV co-infected patients in Phnom Penh. These data suggest that the prevalence of DR TB in Cambodia may be higher than previously recognised, particularly amongst HIV-infected patients. Additional prevalence studies are needed. This study also illustrates the feasibility and utility of analysis of non-respiratory specimens in the diagnosis of TB, even in low-resource settings, and suggests that extra-pulmonary specimens should be included in TB diagnostic algorithms.

Tuberculosis (TB) has re-emerged as one of the most significant infectious diseases of modern times, after more than a century of declining incidence and mortality (1). TB was responsible for an estimated 1.3 million deaths worldwide in 2012 (2). The emergence of human immunodeficiency virus (HIV) infection has driven the resurgence of TB, particularly in resource-limited countries (2, 3), where the ability to diagnose, treat, and control either disease may be limited. These problems are compounded by the emergence of drug-resistant tuberculosis (DR TB), the extent of which is often unknown in a population and undiagnosed in individuals (4). Of particular concern is multidrug-resistant tuberculosis (MDR TB), defined as resistance to at least rifampicin and isoniazid, which requires prolonged, poorly tolerated, and expensive treatment.

The World Health Organization (WHO) defines Cambodia as a country with a high burden of TB (2). In 2012, the estimated prevalence of TB was 764 per 100,000 population (range 645–892 per 100,000). HIV co-infection was sought in 80% of notified cases of TB and found in 4% (2). The last Cambodian national drug resistance survey is more than 10 years old and reported no cases of MDR TB amongst new cases and a prevalence of 3.6% (95% CI 1.0–9.0) amongst previously treated cases (5). The WHO estimated that MDR TB comprised 1.4% of new cases and 11% of previously treated cases of TB in Cambodia in 2012. This equated to 386 expected new and previously treated cases of MDR pulmonary TB, although only 102 were notified that year (2, 4).

There are few specific data on the prevalence of DR TB in HIV-infected patients in Cambodia. The CAMELIA study (6), a large randomised clinical trial investigating the mortality impact of early antiretroviral therapy (ART) in TB–HIV co-infected patients, enrolled HIV-infected adults with CD4 count <200/mm^3^ and a first episode of pulmonary or extra-pulmonary TB. In this study, 13 patients had MDR TB (13/645, 2%) and 73 patients had isoniazid resistance (73/645, 11%). A laboratory-based study conducted in Phnom Penh, Cambodia, found an MDR TB prevalence of 5.5% in 98 samples from HIV-infected patients, but case definitions were not assigned to the drug resistance patterns (7). A 2002 survey of 441 HIV-infected patients in Phnom Penh identified culture-positive pulmonary TB in 9%; isoniazid resistance was identified in 15%, and no MDR TB was found (8).

In this paper, we report the proportion of drug-resistant pulmonary and extra-pulmonary TB in a cohort of HIV-infected patients at a national referral hospital in Phnom Penh.

## Methods

### Study design and population

This is a retrospective observational cohort study of consecutive *Mycobacterium tuberculosis* culture-positive TB–HIV co-infected inpatients and outpatients managed by the Infectious Diseases Unit of Preah Bat Norodom Sihanouk Hospital (Khmer-Soviet Friendship Hospital) over 2 years (November 2007 to November 2009). Preah Bat Norodom Sihanouk Hospital is a large public hospital in Phnom Penh providing healthcare to a generally impoverished population; 58% of the cohort earned less than 200,000 riels (around USD50 per month) [MSF internal report, Phnom Penh]. Médecins Sans Frontières (MSF) supported a TB–HIV programme from 2005 until 2010 and an MDR TB–HIV programme from 2007 until 2009.

Patients enrolled in the HIV cohort were referred from a variety of sources (Table 1) [MSF internal report, Phnom Penh 2007]. After February 2009, in an attempt to limit growth of the cohort prior to integration with the Cambodian National HIV programme, the 233 new HIV patients included in the cohort were mostly inpatient referrals who were eligible for anti-retroviral therapy (ART) [MSF internal report, Phnom Penh 2009].

**Table 1:** Source of referral of new patients included in HIV cohort (2007)

Source of referral	Number of patients, *n*=824 (%)	Inpatients (%)	Outpatients (%)
VCT centre	207 (25.1)	30 (14.4)	177 (85.6)
Respiratory department	141 (17)	17 (12.1)	124 (87.9)
Self-referral via emergency department	174 (21.1)	164 (94.3)	10 (5.7)
Other hospital departments	8 (1.0)	8	0
Family/contact of existing patient	110 (13.3)	1 (0.9)	109 (99.1)
Private doctor	46 (5.6)	15 (32.6)	31 (67.4)
Transfer from another HIV cohort	95 (11.5)	50 (52.6)	45 (47.4)
Prisoner	18 (2.2)	2	16
Other	25 (3.0)	4	21

VCT: Voluntary Counselling and Testing centre.

Most patients were from Phnom Penh (61.6%); 26.4% were from the surrounding provinces of Kandal, Kampong Speu, Prey Veng, Kampong Cham, and Kampong Chhnang. The remaining 12% were from distant provinces [MSF internal report, Phnom Penh 2008].

From 22 November 2007, culture for *M. tuberculosis* and drug susceptibility testing (DST) was routinely performed on specimens from HIV-infected patients aged 15 years and above, suspected of having either pulmonary or extra-pulmonary TB. Patients were suspected of having pulmonary TB if they had had a cough for more than 2 weeks (9). In addition, patients with unexplained fever or weight loss were suspected of having pulmonary and/or extra-pulmonary TB, as were patients with specific signs or symptoms (e.g. lymphadenopathy) consistent with extra-pulmonary TB.

Patients diagnosed with TB were assigned case definitions according to standard WHO definitions (10). WHO definitions describing treatment history were also applied: ‘new cases’ or previously treated cases (either ‘relapsed’, ‘failed’, or ‘interrupted treatment’) (10). Patients who did not fit the above definitions were described as ‘other’ cases. Descriptions of *M. tuberculosis* drug resistance were also assigned according to WHO definitions (11).

### Laboratory procedures

The decision to test a patient suspected of having TB was made according to MSF protocols for the diagnosis of pulmonary TB in HIV-infected patients, based on the symptoms listed above (9); in cases of extra-pulmonary TB or where there was a strong suspicion of pulmonary TB not fulfilling the criteria of protocols, the treating clinician's discretion and other published MSF guidance was also used to inform testing decisions (9). Where pulmonary TB was suspected, two sputum samples were collected and sent to the Pasteur Institute in Phnom Penh for culture and DST. Gastric lavage was used if the patient could not produce sputum, and bronchoalveolar lavage (BAL) could also be performed. In the case of suspected extra-pulmonary TB, specimens from other sites (e.g. aspirated pus) were sent for microscopy and culture.

Smear microscopy was performed using a conventional fluorescence microscopy after specimen concentration using high-speed centrifugation. *M. tuberculosis* was cultured on Lowenstein–Jensen solid media and liquid MGIT (mycobacterial growth indicator tube) media, using the BD MGIT reading manual method prior to May 2009 and the MGIT Bactec 960 method thereafter. DST was performed for any positive *M. tuberculosis* isolate against first-line drugs (rifampicin, isoniazid, ethambutol, and streptomycin), using MGIT. Patients with positive non-tuberculous mycobacterial cultures or mixed cultures were excluded from this analysis.

### Data collection and statistical analysis

The authors (GW, SB, and TD), who were also treating clinicians over the study period, collected clinical information from the hospital and laboratory records of patients with positive *M. tuberculosis* cultures. Data initially collected included gender, geographic origin, and source of entry in to the cohort, site of TB, past TB treatment history, type of clinical specimen, smear status, and drug susceptibility results. Anonymised data were entered into an Excel spreadsheet using the reference number assigned to each patient on enrolment in to the HIV cohort. At a later date, additional data were collected by one of the authors (GW) from the MSF HIV programme database (FUCHIA) using the anonymised reference numbers. No identifiable patient details were available from FUCHIA. These data included age, CD4 count, other opportunistic infection, start date of antiretroviral treatment and TB treatment, and date of entry in to the HIV cohort. In some instances, not all additional data were complete, and patients’ hospital records were no longer available for perusal.

This paper is descriptive and reports data used for clinical decision making, reflecting a routine standard of care in this programme. The data were collected by clinicians involved in patients’ care and all personal data were immediately anonymised before further analysis or later data collection occurred. No intervention occurred based on the anonymised data. Therefore, ethics committee approval was not sought and patients did not sign an informed consent.

The characteristics of patients and specimens from culture-confirmed TB patients are presented. The proportions of each type of drug resistance were calculated for new and previously treated cases, and were compared using Chi-squared tests (Stata Data Analysis and Statistical Software, Version 12.0).

## Results

**Fig. 1:**
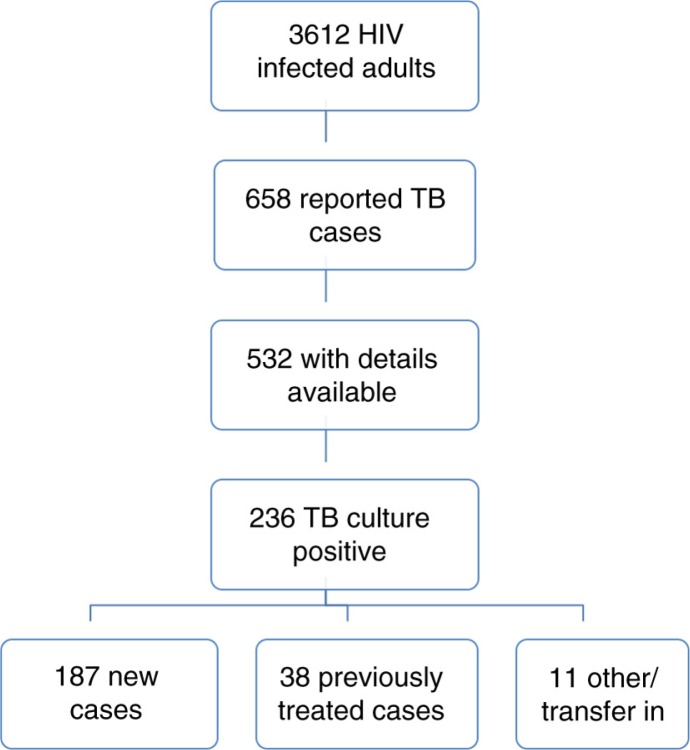
TB cases amongst HIV-infected patients.

Between 1 November 2007 and 30 November 2009, 658 cases of TB were reported in the programme's cohort of 3,612 HIV-infected adult patients (18.2%) who received inpatient and outpatient care over the study period. *M. tuberculosis* infection was confirmed by culture in 236 patients (236/658 cases, 35.9%). Case definitions of the 236 culture-positive patients were as follows: 187 new cases (187/236, 79.2%) and 38 previously treated cases (38/236, 16.1%): 26 relapsed (26/38, 68.4%), 6 who had failed treatment (6/38, 15.8%) and 6 who had interrupted previous TB treatment (6/38, 15.8%). The remainder with either transferred in from another cohort or ‘other’ case definitions (Fig. 1).

Table 2 describes characteristics of the patients with culture-positive pulmonary and extra-pulmonary TB. The majority of patients were male (152/236 patients, 64.4%) with a low median CD4 count (52 cells/mm^3^ **)**; 12.2% also had other opportunistic infections at TB diagnosis. More than half of patients had extra-pulmonary involvement (130/236 patients, 55.1%), indicating widespread disease.

**Table 2:** Patient characteristics at the time of TB diagnosis

	Number of patients^a^	Characteristics, *n* (%)
Male gender	236	152 (64.4)
Median age (years)	196	38 (range 17–63)
Geographic origin	236	
Phnom Penh		125 (53.0)
Outside Phnom Penh		80 (33.9)
Unknown		31 (13.1)
Source of referral	236	
Self-referral via emergency department		97 (41.1)
Unknown		31 (13.1)
Other hospital/cohort		22 (9.3)
Other		18 (7.6)
Voluntary Counselling and Testing centre		17 (7.2)
Respiratory department KSF Hospital		16 (6.8)
Prison		14 (5.9)
Family/contact tracing		8 (3.4)
Other department KSF Hospital		7 (3.0)
Private patient		5 (2.1)
Other NGO		1 (0.3)
Median CD4 count at TB diagnosis (/mm^3^)	160	52 (range 1–703)
On ART at time of TB diagnosis	164	101 (61.6)
TB diagnosed within 60 days after starting ART	101	49 (48.5)
Started ART after TB diagnosis	164	63 (38.4)
Started ART within 2 months after TB treatment	63	43 (68.3)
Opportunistic infection (not TB) at TB diagnosis	196	24 (12.2)
Pulmonary TB	236	204 (85.6)
With extra-pulmonary involvement	204	102 (50)
Extra-pulmonary TB (EPTB)	236	28 (11.9)
Lymph nodes	28	12 (42.9)
Meningitis	28	8 (28.6)
Pleural	28	2 (7.1)

a For whom data were available – not all data were available for all patients. KSF Hospital: Khmer Soviet Friendship Hospital; ART: antiretroviral therapy; TB: tuberculosis. Four patients had TB at an unknown site.

Only 61.6% were on ART at the time of TB diagnosis, despite the low median CD4 count. Of those not on ART at the time of TB diagnosis (63/164 patients, 38.4%), 50 patients (79.4%) recorded a date of enrolment in to the HIV cohort within 1 month before starting TB treatment, indicating that the diagnosis of TB was the precipitant for enrolment in to the HIV cohort, and possibly the reason for the diagnosis of HIV. Of those on ART, TB was diagnosed within 60 days after starting ART in 48.5% of those patients, possibly indicating that TB was a manifestation of an immune reconstitution inflammatory syndrome (IRIS), or that ART was started concurrently with, or soon after, TB treatment.

One-third of the TB–HIV cohort came from outside Phnom Penh and patients were referred by a variety of services, both inpatient from the emergency department and other hospital departments (60.2%), and outpatient (18.6%), giving a mix of acutely unwell and ambulatory patients. After February 2009, patient enrolment into the HIV cohort (but not the HIV–TB cohort) was limited, and most of the 233 new HIV patients were referred from inpatient services. After February 2009, 43 patients were included in the TB–HIV cohort; before February 2009, 193 patients were included. There was no statistically significant difference in the proportions of DR TB seen in patients enrolled before and after February 2009.

Table 3 shows the proportion of drug resistance according to case definition. In total, 82 patients (82/236, 34.7%) had infection with an *M. tuberculosis* isolate exhibiting some form of drug resistance. MDR TB was identified in 8.1% (19/236 patients). Information on treatment was available for 14/19 patients (73.7%) with MDR TB. Three patients (3/19, 15.8%) were diagnosed with MDR TB after death and two others died while on MDR TB treatment (5/19 total deaths, 26.3%). One declined treatment. Three received empiric MDR TB treatment before culture results were available. Seven received MDR TB treatment after culture results; the median delay in treatment initiation was 14 days (range 6–151 days).

**Table 3:** *M. tuberculosis* resistance in 236 HIV-infected patients

	Resistance pattern					
	
Case definition	Fully susceptible	MDR TB	R or RS resistance	H or HS resistance	S mono-resistance	Total patients
New case	132 (70.6)	7 (3.7)	3 (1.6)	26 (13.9)	19 (10.2)	187 (100)
Previously treated case	14 (36.8)	11 (28.9)	7 (18.4)	3 (7.9)	3 (7.9)	38 (100)
*P* a	<0.001	<0.001	<0.001	0.43	–	–
Other case definition	8	1	1	1	0	11 (100)
Sub-totals	154 (65.3)	19 (8.1)	11 (4.7)	30 (12.7)	22 (9.3)	236 (100)
Total	154 (65.3)			82 (34.7)		236 (100)

a The difference between proportion of resistance in new versus previously treated cases. MDR TB: multidrug resistant TB; R or RS: resistance to rifampicin or rifampicin and streptomycin; H or HS: resistance to isoniazid or isoniazid and streptomycin; S: streptomycin resistance. No ethambutol resistance detected. Pyrazinamide not tested.

The proportion of MDR TB was higher amongst previously treated cases than new cases [11/38 previously treated patients (28.9%) vs. 7/187 new patients (3.7%); *p*<0.001], as was the proportion of rifampicin mono-resistance and rifampicin with streptomycin resistance [7/38 patients (18.4%) vs. 3/187 patients (1.6%); *p<*0.001]. Of the 19 patients with MDR TB, seven (7/19, 36.8%) had not previously received TB treatment.

Patients with sputum-smear negative pulmonary TB were more likely to culture a fully susceptible organism than those with smear-positive pulmonary TB [113/153 patients (73.9%) vs. 42/47 patients (89.4%); *p*=0.028], and those with pulmonary TB were more likely to culture a fully susceptible organism than those with extra-pulmonary TB [155/204 patients (76.0%) vs. 16/28 patients (57.1%); *p*=0.041]. Otherwise there was no significant difference between site of TB, smear status, and the identification of resistant organisms in culture.

Table 4 shows the proportion of drug-resistant TB according to HIV demographics and HIV disease profiles. There was no statistically significant difference in the proportion of susceptible or drug-resistant TB between men and women. Younger patients (15–30 years) were more likely to have isoniazid-resistant TB than middle-aged (31–49 years) patients [14/62 patients (22.6%) vs. 7/117 patients (6.0%); *p*=0.003] and were less likely to have fully susceptible or streptomycin mono-resistant TB [40/62 patients (64.5%) vs. 95/117 patients (81.2%); *p*=0.018]; otherwise there was no association between age and drug resistance. Similarly, there were no statistically significant differences in TB drug resistance between patients from Phnom Penh and patients from elsewhere, and those with different CD4 count levels. MDR TB occurred more in patients with HIV diagnosed over a year prior to the TB diagnosis (11/58, 19%) than those with a more recent diagnosis of HIV infection (6/138, 4.3%; *p*=0.0018). Patients who were already on ART were more likely to have MDR TB (15/101, 14.9%) than those who were not on ART at the time of TB diagnosis (2/63, 3.2%; *p*=0.018), and less likely to have susceptible TB [68/101 (67.3%) vs. 55/63 (87.3%); *p*=0.0051].

**Table 4:** Patterns of TB drug resistance in HIV patients by patient characteristics

		Pattern of TB resistance			
		
Patient characteristics	Number of patients^a^ (%)	Fully susceptible/only streptomycin resistant	MDR	R/RS	H/HS
Gender	236	176 (74.6)	19 (8.1)	11 (4.7)	30 (12.7)
Male	152	115 (75.7)	13 (8.6)	6 (3.9)	18 (11.8)
Female	84	61 (72.6)	6 (7.1)	5 (6.0)	12 (14.3)
Age (years)	196	150 (76.5)	17 (8.7)	7 (3.6)	22 (11.2)
15–30	62	**40 (64.5)^b^**	5 (8.1)	3 (4.8)	**14 (22.6)**
31–49	117	**95 (81.2)**	11 (9.4)	4 (3.4)	**7 (6.0)**
≥50	17	15	1 (5.9)	0	1
Place of origin	236	176 (74.6)	19 (8.1)	11 (4.7)	30 (12.7)
Phnom Penh	125	96 (76.8)	11 (8.8)	3 (2.4)	15 (12)
Outside Phnom Penh	80	58 (72.5)	7 (8.8)	6 (7.5)	9 (11.3)
Unknown	31	22 (73.3)	1 (3.2)	2 (6.5)	6 (19.4)
CD4 count at TB diagnosis (/mm^3^)	160	124 (77.5)	14 (8.8)	3 (1.9)	19 (11.9)
<50	76	61 (80.3)	5 (6.6)	2 (2.6)	8 (10.5)
50–200	54	41 (75.9)	4 (7.4)	1 (1.9)	8 (14.8)
>200	30	22 (73.3)	5 (16.7)	0	3 (10.0)
HIV duration prior to TB diagnosis	196	150 (76.5)	17 (8.7)	7 (3.6)	22 (11.4)
<1 year (or concurrent HIV diagnosis)	138	109 (79.0)	**6 (4.3)**	4 (2.9)	19 (13.8)
>1 year prior	58	41 (70.7)	**11 (19.0)**	3 (5.2)	3 (5.2)
Antiretroviral therapy (ART)	164	123 (75.0)	17 (10.4)	7 (4.3)	17 (10.4)
On ART at TB diagnosis	101	**68 (67.3)**	**15 (14.9)**	7 (6.9)	11 (10.9)
TB diagnosed within 60 days after starting ART	49	36 (73.5)	4 (8.2)	2 (4.1)	7 (14.3)
Not on ART at TB diagnosis	63	**55 (87.3)**	**2 (3.2)**	0	6 (9.5)
Started ART within 60 days after TB treatment	43	36 (83.7)	2 (4.7)	0	5 (11.6)

a For whom data available; not all data were available for all patients;

b statistically significant differences are highlighted in bold (see Results).

There were 284 positive specimens recorded from 236 patients. Respiratory specimens were the most common (204/284, 72%): 189 spontaneously produced sputa, 11 gastric fluid aspirates, and 4 BAL specimens. Of the respiratory specimens, 149 (73%) were smear-positive, 48 (24%) were smear-negative, and the smear status of seven was unknown. Other non-respiratory specimens (67/284, 23.6%) included pus, cerebrospinal fluid, faeces, pleural fluid, synovial fluid, blood, urine, pericardial, and peritoneal fluid. In 13 cases (13/284, 4.6%), the specimen type was not recorded. TB was diagnosed from culture of 67 non-respiratory specimens (67/284, 23.6%), with DR TB being identified from culture of 27/67 non-respiratory specimens (40.3%) and MDR TB from 7/67 (10.4%) non-respiratory specimens (10.4%).

## Discussion

This study describes resistance to first-line TB drugs in a cohort of culture-positive TB–HIV co-infected adults at a large public hospital in Phnom Penh. We found that 34.7% of patients were infected with an *M. tuberculosis* isolate exhibiting some form of drug resistance. MDR TB was found in 8.1%; 36.8% of the MDR TB patients had not previously received TB treatment, which implies that MDR TB is being transmitted in this population. In addition, 15.5% of new patients and 26.3% of previously treated patients had rifampicin or isoniazid resistance. These resistance patterns also require specific treatment and confer risk for amplification of resistance if treated with standard TB treatment.

The proportion of MDR TB in this cohort is higher than previous surveys and WHO estimates of MDR TB in Cambodia would suggest (2, 5). However, there are few data on the prevalence of DR TB in the Cambodian population. The last formal Cambodian TB drug resistance survey was performed in 2000–2001 (5) and found a prevalence of any drug resistance of 10.1% amongst new cases and 16.6% amongst previously treated cases. The prevalence of HIV infection in these patients was not known.

Little is known about the prevalence of DR TB amongst HIV-infected patients in Cambodia. The CAMELIA study (6) reports MDR TB in 2% and isoniazid-resistant TB in 11% of 645 HIV-infected patients. A laboratory-based study from Phnom Penh published in 2009 found an MDR TB prevalence of 5.5% in 98 samples from HIV-infected patients (7); however, clinical data and case definitions were not available for these patients.

The comparatively high prevalence of DR TB in our cohort (34.7%) may reflect an increasing incidence of DR TB in Cambodian HIV-infected patients, and possibly in the general population. However, our results, from a specific population of patients in a tertiary hospital (who are perhaps more complex than other patients), may not be able to be generalised to the population as a whole.

These data suggest the need for another formal prevalence study in Cambodia. Knowledge of the prevalence of DR TB in an HIV-infected population, and the identification of DR TB in an individual HIV-infected patient, is crucial for a number of reasons.

On an individual level, the treatment for both DR TB (particularly MDR TB) and HIV is prolonged, expensive, and complicated compared with the treatment for susceptible TB and HIV co-infection. The complexities of treatment increase the risk of patient harm (including the risk of treatment failure) to the patient through drug interactions, drug intolerance, and non-compliance. In addition, treatment outcomes appear to be poorer for HIV-infected patients who also have DR TB. Mortality rates in MDR TB and HIV co-infected people are higher than for HIV-infected patients with susceptible TB (12).

The co-incidence of DR TB and HIV may also have implications for TB control programmes.

The high prevalence of DR TB in this study may indicate that those with HIV infection are at increased risk of acquiring DR TB. HIV infection is the primary driver in the global spread of TB (3, 13), being the greatest risk factor, after infection with *M. tuberculosis* itself, for progression to active TB (2, 13). Although large surveillance projects have been unable to support an association between HIV infection and TB drug resistance (4, 14), data from certain areas may suggest a link. Outbreaks of multi-drug- and extensively-drug-resistant TB in institutions have been associated with HIV infection in low-resource (15) and high-resource countries (16), and in a recent national survey involving hospitals and health centres in Swaziland, HIV infection was independently associated with the risk of MDR TB (17).

The rapid and accurate diagnosis of DR TB, particularly MDR TB, is essential on both an individual and a programmatic level. Current methods of DST used in many countries do not give useful results for many weeks. The Cambodian National TB programme and the WHO recommend DST for all previously treated patients with TB; in HIV-infected TB suspects, the WHO recommends using the XpertMTB/RIF genotypic assay (2). DST by phenotypic methods is not widely available in laboratories in Cambodia, and the XpertMTB/RIF is usually only available in a small number of private laboratories. Laboratories with the ability to do DST or molecular testing are usually situated in major centres and are not generally accessible to those in remote areas.

The rapidity with which genotypic methods are able to identify MDR TB is their major advantage, allowing timely initiation of appropriate MDR TB treatment (18–20) . Delay in appropriate TB treatment in HIV-infected patients with DR TB has been shown to increase the risk of death (21). In our study, one-quarter 26.3% of the patients with MDR TB were dead by at the time, or soon after, the results of phenotypic DST were received. The delay in starting appropriate MDR TB treatment probably contributed to their deaths, and can also contribute to the on-going transmission of DR TB (20). There are challenges in the implementation of rapid genotypic tests in many high burden countries (22).

This study has some limitations, the most important being the retrospective nature of the analysis. Despite protocols for the diagnosis of TB, many patients were started on treatment empirically, without culture confirmation. It is possible that the culture-positive patients received more systematic culture investigation because they were more complicated or unwell. This may have falsely elevated the proportion of DR TB. However, the large, representative cohort of HIV-infected patients from whom this study population was drawn included a substantial proportion of outpatients, who are generally less complicated.

Another source of bias may be the fact that the study occurred in a national referral hospital, where the patients may have been more complicated because of referral patterns. However, patients were referred from many different sources and geographic locations. No significant difference was found in the proportion of DR TB in patients from Phnom Penh compared with patients from other areas, suggesting that the study cohort was not unduly biased by the receipt of sick and/or complicated patients from other cohorts or hospitals.

The history of previous treatment may have been inaccurate in some cases: 11 patients (4.7%) did not have an assigned case definition, and it is possible that the number of ‘new cases’ was over-estimated, as ‘new case’ may have been the default case definition when treatment history was unclear. However, all 19 MDR TB cases were reviewed by the authors, meaning that these case definitions are correct. A system was in place to collect and record all the results of specimens sent for TB culture (both positive and negative), but it is also possible that some may have been missed.

A strength of this study is the inclusion of patients with smear-negative pulmonary and extra-pulmonary TB. Most DR TB prevalence studies focus on the large sub-group of smear-positive pulmonary TB, and previous studies in Cambodia have been either laboratory-based (7), or clinical trials not specifically designed to describe DR TB prevalence in HIV-infected patients (6, 8). HIV-infected people are more likely to develop smear-negative and extra-pulmonary TB than those without HIV infection (23), and the exclusion of these sub-groups from descriptions of DR TB in HIV-infected patients is likely to lead to an under-estimate of DR TB prevalence. In addition, culture, and thus confirmation, of DR TB in HIV-infected patients with smear-negative pulmonary TB and extra-pulmonary TB is difficult in many clinical settings, leading to further under-estimates. In this cohort of HIV-infected patients, TB was confirmed by culture of extra-pulmonary specimens in 23.6%; in addition, 40.3% of those non-respiratory specimens identified DR TB. This demonstrates that analysis of extra-pulmonary specimens is feasible, and necessary, in the diagnosis and appropriate management of TB and DR TB in resource-limited settings. The inclusion of all sites of TB infection in our study allows us to provide a general picture of TB–HIV co-infection.

## Conclusions

There is a paucity of data on TB drug resistance in Cambodia, particularly amongst HIV-infected patients. Our data show a high proportion of drug resistance amongst new and previously treated patients with TB–HIV co-infection in a referral hospital in Phnom Penh. This has implications for treatment on an individual level, and for the detection and control of TB on a population level, for which additional surveillance studies are required. In particular, these data reinforce the urgent need for accessible means of rapidly identifying and treating drug-resistant TB for high-burden and resource-limited countries.
